# Unbiased bootstrap error estimation for linear discriminant analysis

**DOI:** 10.1186/s13637-014-0015-0

**Published:** 2014-10-03

**Authors:** Thang Vu, Chao Sima, Ulisses M Braga-Neto, Edward R Dougherty

**Affiliations:** 1 Department of Electrical and Computer Engineering, Texas A&M University, 3128 TAMU, College Station, 77843 TX USA; 2 Center for Bioinformatics and Genomic Systems Engineering, Texas A&M University, 101 Gateway, Suite A, College Station, 77845 TX USA

**Keywords:** Bootstrap, Error estimation, Bias, Linear discriminant analysis, Gene expression classification

## Abstract

Convex bootstrap error estimation is a popular tool for classifier error estimation in gene expression studies. A basic question is how to determine the weight for the convex combination between the basic bootstrap estimator and the resubstitution estimator such that the resulting estimator is unbiased at finite sample sizes. The well-known 0.632 bootstrap error estimator uses asymptotic arguments to propose a fixed 0.632 weight, whereas the more recent 0.632+ bootstrap error estimator attempts to set the weight adaptively. In this paper, we study the finite sample problem in the case of linear discriminant analysis under Gaussian populations. We derive exact expressions for the weight that guarantee unbiasedness of the convex bootstrap error estimator in the univariate and multivariate cases, without making asymptotic simplifications. Using exact computation in the univariate case and an accurate approximation in the multivariate case, we obtain the required weight and show that it can deviate significantly from the constant 0.632 weight, depending on the sample size and Bayes error for the problem. The methodology is illustrated by application on data from a well-known cancer classification study.

**Electronic supplementary material:**

The online version of this article (doi:10.1186/s13637-014-0015-0) contains supplementary material, which is available to authorized users.

## 1Introduction

The bootstrap method [1]–[7] has been used in a wide range of statistical problems. The asymptotic behavior of bootstrap has been studied [8]–[11], while small-sample properties have been studied under simplifying assumptions, such as considering the estimator based on all possible bootstrap samples (the ‘complete’ bootstrap) [12]–[14]. The small-sample properties of the usual bootstrap are not well understood, in particular when it comes to estimating the error rates of classification rules [15],[16].

There has been, on the other hand, interest in the application of bootstrap to error estimation in classification problems and, in particular, gene expression classification studies [17]–[20]. Of particular interest is the issue of classifier error estimation [21],[22]. Bootstrap methods have generally been shown to outperform more traditional error estimation techniques, such as resubstitution and cross-validation, in terms of root-mean-square (RMS) error [4],[5],[7],[23]–[35]. Bootstrap error estimation is typically performed via a convex combination of the (generally) pessimistic basic bootstrap estimator, known as the zero bootstrap, and the (generally) optimistic resubstitution estimator. A basic problem is how to choose the weight that yields an unbiased estimator.

The problem of unbiased convex error estimation was previously considered in [36]–[38] for a convex combination of resubstitution and cross-validation estimators, and in [4],[7],[23] for a combination between resubstitution and the basic bootstrap estimator. In the former case, a fixed suboptimal weight of 0.5 was proposed in [36],[38], while an asymptotic analysis to find the optimal weight was provided in [37]. In the latter case, our case of interest, a fixed suboptimal weight of 0.632 was proposed in [4], leading to the well-known 0.632 bootstrap estimator, while in [7], a suboptimal weight is computed by means of a sample-based procedure, which attempts to counterbalance the effect of overfitting on the bias, leading to the so-called 0.632+ bootstrap error estimator; the problem of finding the optimal weight for finite sample cases was addressed via a numerical approach in [23].

Here, we determine the optimal weight for finite sample cases analytically, in the case of linear discriminant analysis under Gaussian populations. In the univariate case, no other assumptions are made. In the multivariate case, it is assumed that the populations are homoskedastic and that the common covariance matrix is known and used in the discriminant. In either case, no simplifications are introduced to the bootstrap error estimator; it is the usual one, based on a finite number of random bootstrap samples.

The analysis in this paper follows in the steps of previous papers that have provided analytical representations for the moments of error-estimator distributions [39],[40]. In the univariate case, exact expressions are given for the expectation of the zero bootstrap error estimator, in the general heteroskedastic (general-variance) Gaussian case. By using similar expressions for the expected true and resubstitution error [39], this allows the exact calculation of the required weight. In the multivariate case, the expectation of the zero bootstrap error estimator is expressed as a probability involving the ratio of two noncentral chi-square variables, in the homoskedastic Gaussian case, assuming that the true common covariance matrix is used in the discriminant. The resulting expression is exact but necessitates approximation for its numerical computation. This is done in this paper via the Imhof-Pearson three-moment method, which is accurate in small-sample cases [41]. Use of similar expressions for the expected true and resubstitution error [40] then allows the exact calculation of the required weight.

In the homoskedastic case, the required weight for unbiasedness is shown to be a function only of the Bayes error and sample size. Accordingly, plots and tables of the required weight for varying values of Bayes error and sample size are presented; if the Bayes error can be estimated for a problem, this provides a way to obtain the optimal weight to use. In the univariate case, it was observed that as the sample size increases, the optimal weight settles on an asymptotic value of around 0.675, thus slightly over the heuristic value 0.632; by contrast, in the multivariate case (*d*=2), the asymptotic value appears to be strongly dependent on the Bayes error, being as a rule significantly smaller than 0.632, except for very small Bayes error.

This paper is organized as follows. The ‘Bootstrap classification’ section defines linear discriminant analysis as well as its application under bootstrap sampling. The ‘Bootstrap error estimation’ section reviews convex bootstrap error estimation. The ‘Unbiased bootstrap error estimation’ section contains the main theoretical results in the paper, providing the analytical expressions for the computation of the required convex bootstrap weight in the univariate and multivariate cases. The ‘Gene expression classification example’ section contains a demonstration of the usage of the optimal weight in bootstrap error estimation using data from the breast cancer classification study in [42],[43]. Lastly, the ‘Conclusions’ section contains a summary and concluding remarks.

All the proofs are presented in the Appendix.

## 2Bootstrap classification

Classification involves a predictor vector *X*∈*R*^*d*^, also known as a *feature* vector, which represents an individual from one of two populations *Π*_0_ and *Π*_1_ (we consider here only this binary classification problem). The classification problem is to assign *X* correctly to its population of origin. The populations are coded into a discrete *label* *Y*∈{0,1}. Therefore, given a feature vector *X*, classification attempts to predict the corresponding value of the label *Y*. We assume that there is a joint *feature-label distribution* *F*_*XY*_ for the pair (*X*,*Y*) characterizing the classification problem. In particular, it determines the probabilities *c*_0_=*P*(*X*∈*Π*_0_)=*P*(*Y*=0) and *c*_1_=*P*(*X*∈*Π*_1_)=*P*(*Y*=1), which are called the *prior probabilities*.

Given a fixed sample size *n*, the *sample data* is an i.i.d. sample *S*_*n*_={(*X*_1_,*Y*_1_),…,(*X*_*n*_,*Y*_*n*_)} from *F*_*XY*_. The population-specific sample sizes are given by $n_{0}=\overset{n}{\underset{i=1}{\sum }}I_{Y_{i}=0}$ and $n_{1}=\overset{n}{\underset{i=1}{\sum }}I_{Y_{i}=1}=n-n_{0}$, which are random variables, with *n*_0_∼Binomial(*n*,*c*_0_) and *n*_1_∼Binomial(*n*,*c*_1_). When we need to emphasize that *n*_0_ and *n*_1_ are random variables, we will use capital letters *N*_0_ and *N*_1_, respectively. This sampling design, which is the most commonly found one in contemporary pattern recognition, is known as *mixture sampling*[44].

A *classification rule* *Ψ*_*n*_ is used to map the training data *S*_*n*_ into a designed classifier *ψ*_*n*_=*Ψ*_*n*_(*S*_*n*_), where *ψ*_*n*_ is a function taking on values in the set {0,1}, such that *X* is assigned to population *Π*_0_ or *Π*_1_ according to whether *ψ*_*n*_(*X*)=0 or 1, respectively. The *classification error rate* *ε*_*n*_ of classifier *ψ*_*n*_ is the probability that the assignment is erroneous:

1 $$\;\begin{array}{cc} \varepsilon_{n} & =c_{0}\;P(\psi_{n}(X)=1|Y=0)+c_{1}\;P(\psi_{n}(X)=0|Y=1)\\ \overset{\text{def}}{=}c_{0}\;\varepsilon_{n}^{0}+c_{1}\;\varepsilon_{n}^{1}, \end{array}$$

where (*X*,*Y*) is an independent test point and $\varepsilon_{n}^{i}=P(\psi_{n}(X)=1-i|Y=i)$ is the error rate specific to population *Π*_*i*_, for *i*=0,1. Since the training set *S*_*n*_ is random, *ε*_*n*_ is a random variable, with *expected classification error rate* *E*[ *ε*_*n*_]; this gives the average performance over all possible training sets *S*_*n*_, for fixed sample size *n*.

*Linear discriminant analysis* (LDA) employs Anderson’s *W* discriminant [45], which is defined as follows:

2 $$W(X)\;=\;{\left(X-\frac{{\hat{\mu }}_{0}+{\hat{\mu }}_{1}}{2}\right)}^{T}\Sigma^{-1}\left({\hat{\mu }}_{0}-{\hat{\mu }}_{1}\right)$$

where

3 $$\begin{array}{cc} {\hat{\mu }}_{0} & \;=\;\frac{1}{n_{0}}\overset{n}{\underset{i=1}{\sum }}X_{i}I_{Y_{i}=0}\;\text{and}\;{\hat{\mu }}_{1}\;=\;\frac{1}{n_{1}}\overset{n}{\underset{i=1}{\sum }}X_{i}I_{Y_{i}=1} \end{array}$$

are the sample means relative to each population, and *Σ* is a matrix, which can be either (1) the true common covariance matrix of the populations, assuming it is known (this is the approach followed, for example, in [39],[40],[46]), or (2) the sample covariance matrix based on the pooled sample *S*_*n*_, which leads to the general LDA case. In this paper, we will assume case (1) throughout.

The corresponding LDA classifier is given by

4 $$\psi_{n}(X)=\left\{\begin{array}{cc} 1\;,\; & \text{if}\;W(X)<0\\ 0\;,\; & \text{if}\;W(X)\geq 0 \end{array}\right.\;,$$

that is, the sign of *W*(*X*) determines the classification of *X*.

A *bootstrap sample*$S_{n}^{*}$ contains *n* instances drawn uniformly, with replacement, from *S*_*n*_. Hence, some of the instances in *S*_*n*_ may appear multiple times in $S_{n}^{*}$, whereas others may not appear at all. Let *C* be a vector of size *n*, where the *i* th component *C*(*i*) equals the number of appearances in $S_{n}^{*}$ of the *i* th instance in *S*_*n*_. The vector *C* will be referred to as a *bootstrap vector*.

For a given *S*_*n*_, the vector *C* uniquely determines a bootstrap sample $S_{n}^{*}$, which we denote by $S_{n}^{C}$. Note that the original sample itself is included: if $C=(1,\ldots ,1)\overset{\text{def}}{=}1_{n}$, then $S_{n}^{C}=S_{n}$, since each original instance appears once in the bootstrap sample. Note also that the number of distinct bootstrap samples, i.e., values for *C*, is equal to 2n−1n; even for small *n*, this is a large number. For example, the total number of possible bootstrap samples of size *n*=20 is larger than 6.8×10^10^.

The vector *C* has a multinomial distribution with parameters (*n*,1/*n*,…,1/*n*),

5 $$\;P(C=(i_{1},\ldots ,i_{n}))\;=\;\frac{1}{n^{n}}\;\frac{n!}{i_{1}!\cdots i_{n}!}\;,\;i_{1}\;+\cdots +\;i_{n}=n\;.$$

Starting from a classification rule *Ψ*_*n*_, one may design a classifier $\psi_{n}^{C}=\Psi_{n}(S^{C})$ on a bootstrap training set *S*^*C*^. Its classification error $\varepsilon_{n}^{C}$ is given as in (1), namely, $\varepsilon_{n}^{C}=c_{0}\varepsilon_{n}^{C,0}+c_{1}\varepsilon_{n}^{C,1}$ where $\varepsilon_{n}^{C,i}=P(\psi_{n}^{C}(X)=1-i|Y=i)$ is the error rate specific to population *Π*_*i*_, for *i*=0,1. In this paper, we apply this scheme to the LDA classification rule defined previously. Notice the distinction between a bootstrap LDA classifier and a ‘bagged’ (bootstrap-aggregated) LDA classifier [47],[48]; these correspond to distinct classification rules. The bootstrap LDA classifier is employed here as an auxiliary tool to analyze the problem of unbiased bootstrap error estimation for the plain LDA classifier.

## 3Bootstrap error estimation

Since the feature-label distribution is typically unknown, the classification error rate *ε*_*n*_ has to be estimated by a sample-based statistic ${\hat{\varepsilon }}_{n}$, commonly referred to as an *error estimator*. Data in practice are often limited, and the training sample *S*_*n*_ has to be used for both designing the classifier *ψ*_*n*_ and as the basis for the error estimator ${\hat{\varepsilon }}_{n}$. The simplest and fastest way to estimate the error of a designed classifier *ψ*_*n*_ is to compute its error on the sample data itself:

6 $${\hat{\varepsilon }}_{n}^{\;r}\;=\;\frac{1}{n}\overset{n}{\underset{i=1}{\sum }}\left(I_{\psi_{n}(X_{i})=1}\;I_{Y_{i}=0}+I_{\psi_{n}(X_{i})=0}\;I_{Y_{i}=1}\right)\;.$$

This *resubstitution* estimator, or *apparent error*, is often optimistically biased, that is, it is often the case that $\text{Bias}\;\left({\hat{\varepsilon }}_{n}^{\;r}\right)=E\;\left[{\hat{\varepsilon }}_{n}^{\;r}\right]-E[\;\varepsilon_{n}]<0$, though this is not always so. The bias tends to worsen with more complex classification rules [49].

The basic bootstrap error estimator is the *zero bootstrap* error estimator [4], which is introduced next. Given the training data *S*_*n*_, *B* bootstrap samples are randomly drawn from it. Denote the corresponding (random) bootstrap vectors by {*C*_1_,…,*C*_*B*_}. The zero bootstrap error estimator is defined as the average error committed by the *B* bootstrap classifiers on sample points that do not appear in the bootstrap samples:

7 $$\begin{array}{cc} {\hat{\varepsilon }}_{n}^{\;\text{boot}} & =\frac{1}{B}\overset{B}{\underset{i=1}{\sum }}\left[\frac{1}{n(C_{i})}\underset{j:\;C_{i}(j)=0}{\sum }\left(I_{\psi_{n}^{C}(X_{j})=1}\;I_{Y_{j}=0}\right.\right.\\ \;+\left.\left.I_{\psi_{n}^{C}(X_{j})=0}\;I_{Y_{j}=1}\right)\right]\;, \end{array}$$

where *n*(*C*) is the number of zeros in *C*.

The bootstrap zero estimator tends to be pessimistically biased, since the amount of distinct training instances available for designing the classifier is on average (1−*e*^−1^)*n*≈0.632*n*<*n*. Pessimistic bias in an error estimator can be mitigated by forming a convex combination with an optimistic error estimator [23]. In the case of bootstrap error estimation, the standard approach is to form a convex combination of the zero bootstrap with resubstitution,

8 $${\hat{\varepsilon }}_{n}^{\;\text{conv}}\;=\;(1-w)\;{\hat{\varepsilon }}_{n}^{\;r}+w\;{\hat{\varepsilon }}_{n}^{\;\text{boot}}\;.$$

Selecting the appropriate weight *w*=*w*^∗^ leads to an unbiased error estimator, $E[\;{\hat{\varepsilon }}_{n}^{\;\text{conv}}]=E[\;\varepsilon_{n}]$.

In [4], the weight *w* is heuristically set to *w*=0.632 to reflect the average ratio of original training instances that appear in a bootstrap sample. This is known as the *.632 bootstrap estimator*

9 $${\hat{\varepsilon }}_{n}^{\;b632}\;=\;(1-0.632)\;{\hat{\varepsilon }}_{n}^{\;r}+0.632\;{\hat{\varepsilon }}_{n}^{\;\text{boot}}\;,$$

which has been heavily employed in the machine learning field.

## 4Unbiased bootstrap error estimation

The 0.632 bootstrap error estimator reviewed in the previous section is not guaranteed to be unbiased. In this section, we will examine the necessary conditions for setting the weight *w*=*w*^∗^ in (8) to achieve unbiasedness. We will then particularize the analysis to the Gaussian linear discriminant case, where exact expressions for *w*^∗^ will be derived, both in the univariate and multivariate cases.

The bias of the convex estimator in (8) is given by

10 $$E\left[{\hat{\varepsilon }}_{n}^{\;\text{conv}}-\varepsilon_{n}\right]\;=\;(1-w)E\left[{\hat{\varepsilon }}_{n}^{\;r}\right]+\text{wE}\left[{\hat{\varepsilon }}_{n}^{\;\text{boot}}\right]-E\left[\varepsilon_{n}\right]\;.$$

Setting this to zero yields the exact weight

11 $$w^{*}\;=\;\frac{E\left[{\hat{\varepsilon }}_{n}^{\;r}\right]-E\left[\varepsilon_{n}\right]}{E\left[{\hat{\varepsilon }}_{n}^{\;r}\right]-E\left[{\hat{\varepsilon }}_{n}^{\;\text{boot}}\right]}$$

that produces an unbiased error estimator.

Now, applying expectation on both sides of (7) produces

12 $$E\left[{\hat{\varepsilon }}_{n}^{\;\text{boot}}\right]\;=\;\underset{C}{\sum }E\left[\varepsilon_{n}^{\;C}|C\right]\;p(C)\;,$$

where *p*(*C*) is given by (5) and the sum is taken over all possible values of *C* (an efficient procedure for listing all multinomial vectors is provided by the NEXCOM routine given in [50], Chapter 5). Equations (11) and (12) allow the computation of the weight *w*^∗^ given the knowledge of *E*[*ε*_*n*_], $E\left[{\hat{\varepsilon }}_{n}^{\;r}\right]$, and $E\left[\varepsilon_{n}^{\;C}|C\right]$. We will present next exact formulas for these expectations in the case of the LDA classification rule under Gaussian populations.

### 4.1 Univariate case

In the univariate case, the common variance term cancels and the *W* statistic and LDA classifier become greatly simplified, with

13 $$\psi_{n}(X)=\left\{\begin{array}{cc} 1\;,\; & \text{if}\left(X-\frac{{\hat{\mu }}_{0}+{\hat{\mu }}_{1}}{2}\right)({\hat{\mu }}_{0}-{\hat{\mu }}_{1})<0\\ 0\;,\; & \text{otherwise} \end{array}\right.\;.$$

The following functions will be useful. Let *Φ*(*u*)=*P*(*Z*≤*u*) and *Φ*(*u*,*v*;*ρ*)=*P*((*Z*_1_,*Z*_2_)≤(*u*,*v*)), where *Z* is a zero-mean, unit-variance Gaussian random variable, and *Z*_1_, *Z*_2_ are zero-mean, unit-variance random variables that are jointly Gaussian distributed, with correlation coefficient *ρ*.

Assume that population *Π*_*i*_ is distributed as *N*(*μ*_*i*_,*σ*_*i*_), for *i*=0,1, where *σ*_0_≠*σ*_1_ in general.

Under these conditions, John obtained in [39] an exact expression for the expectation of the true classification error for *fixed* sample sizes *n*_0_ and *n*_1_ (this is known as *separate* sampling [44]). John’s result can be written as follows:

14 $$E\left[\varepsilon_{n}^{0}|N_{0}=n_{0}\right]\;=\;\Phi (a,b;\rho_{e})+\Phi (-a,-b;\rho_{e})\;,$$

where

15 $$\begin{array}{cc} a & =\frac{\mu_{1}-\mu_{0}}{\sqrt{\frac{\sigma_{0}^{2}}{n_{0}}+\frac{\sigma_{1}^{2}}{n_{1}}}}\;,\;b\;=\;\frac{\mu_{0}-\mu_{1}}{\sqrt{\left(4+\frac{1}{n_{0}}\right)\sigma_{0}^{2}+\frac{\sigma_{1}^{2}}{n_{1}}}}\;,\\ \rho_{e} & =\frac{\frac{\sigma_{0}^{2}}{n_{0}}-\frac{\sigma_{1}^{2}}{n_{1}}}{\sqrt{\left(\frac{\sigma_{0}^{2}}{n_{0}}+\frac{\sigma_{1}^{2}}{n_{1}}\right)\left(\left(4+\frac{1}{n_{0}}\right)\sigma_{0}^{2}+\frac{\sigma_{1}^{2}}{n_{1}}\right)}}\;. \end{array}$$

The corresponding result for $E[\varepsilon_{n}^{1}|N_{0}=n_{0}]$ is obtained by simply interchanging all indices 0 and 1 in the previous expressions. The expected error rate can then be found by using conditioning and Equation (1):

16 $$\;\begin{array}{cc} E[\varepsilon_{n}] & =\overset{n}{\underset{n_{0}=0}{\sum }}E[\varepsilon_{n}|N_{0}=n_{0}]P(N_{0}=n_{0})\\ =\overset{n}{\underset{n_{0}=0}{\sum }}\left(c_{0}E\left[\varepsilon_{n}^{0}|N_{0}=n_{0}\right]+c_{1}E\left[\varepsilon_{n}^{1}|N_{0}=n_{0}\right]\right)\\ \;\times P(N_{0}=n_{0})\;. \end{array}$$

where

17 P(N0=n0)=nn0c0n0c1n1.

As for resubstitution, Hills provided in [51] exact expressions for the expected error for fixed *n*_0_ and *n*_1_. However, his expression applies only to the case *σ*_0_=*σ*_1_. Theorem 3 in [52] provides a generalization of this result to the case of populations of unequal variances. First, note that

18 $$\begin{array}{c} {\hat{\varepsilon }}_{n}^{\;r}\;=\;\frac{n_{0}}{n}\;{\hat{\varepsilon }}_{n}^{\;r,0}+\frac{n_{1}}{n}\;{\hat{\varepsilon }}_{n}^{\;r,1}\;, \end{array}$$

where

19 $$\begin{array}{cc} {\hat{\varepsilon }}_{n}^{\;r,0} & =\frac{1}{n_{0}}\;\left[\;\overset{n}{\underset{i=1}{\sum }}I_{\psi (X_{i})=1}\;I_{Y_{i}=0}\right]\;\text{and}\\ {\hat{\varepsilon }}_{n}^{\;r,1} & =\frac{1}{n_{1}}\;\left[\;\overset{n}{\underset{i=1}{\sum }}I_{\psi (X_{i})=0}\;I_{Y_{i}=1}\right] \end{array}$$

are the apparent error rates specific to class 0 and 1, respectively. The result in [52] can be written as

20 $$E\left[{\hat{\varepsilon }}_{n}^{\;r,0}|N_{0}=n_{0}\right]\;=\;\Phi (c,d;\rho_{r})+\Phi (-c,-d;\rho_{r})\;,$$

where

21 $$\begin{array}{cc} c & =\frac{\mu_{1}-\mu_{0}}{\sqrt{\frac{\sigma_{0}^{2}}{n_{0}}+\frac{\sigma_{1}^{2}}{n_{1}}}}\;,\;d\;=\;\frac{\mu_{0}-\mu_{1}}{\sqrt{\left(4-\frac{3}{n_{0}}\right)\sigma_{0}^{2}+\frac{\sigma_{1}^{2}}{n_{1}}}}\;,\\ \rho_{r} & =-\frac{\frac{\sigma_{0}^{2}}{n_{0}}+\frac{\sigma_{1}^{2}}{n_{1}}}{\sqrt{\left(\frac{\sigma_{0}^{2}}{n_{0}}+\frac{\sigma_{1}^{2}}{n_{1}}\right)\left(\left(4-\frac{3}{n_{0}}\right)\sigma_{0}^{2}+\frac{\sigma_{1}^{2}}{n_{1}}\right)}}\;. \end{array}$$

The corresponding result for $E[{\hat{\varepsilon }}_{n}^{\;r,1}|N_{0}=n_{0}]$ is obtained by interchanging all indices 0 and 1. The expected resubstitution error rate can then be found by using conditioning and Equation (18):

22 $$\;\begin{array}{cc} E\left[{\hat{\varepsilon }}_{n}^{\;r}\right] & =\overset{n}{\underset{n_{0}=0}{\sum }}E\left[{\hat{\varepsilon }}_{n}^{\;r}|N_{0}=n_{0}\right]P(N_{0}=n_{0})\\ =\overset{n}{\underset{n_{0}=0}{\sum }}\;\left(\frac{n_{0}}{n}E\left[{\hat{\varepsilon }}_{n}^{\;r,0}|N_{0}=n_{0}\right]\;+\;\frac{n_{1}}{n}E\left[{\hat{\varepsilon }}_{n}^{\;r,1}\;|\;N_{0}\;=\;n_{0}\right]\right)\\ \;\times P(N_{0}=n_{0})\;. \end{array}$$

Finally, let us consider the expected bootstrap error. Given *C*, the bootstrap LDA classifier is obtained by replacing ${\hat{\mu }}_{i}$ by ${\hat{\mu }}_{i}^{C}$, *i*=0,1, in (13):

23 $$\psi_{n}^{C}(X)=\left\{\begin{array}{cc} 1\;,\; & \text{if}\;\left(X-\frac{{\hat{\mu }}_{0}^{C}+{\hat{\mu }}_{1}^{C}}{2}\right)\left({\hat{\mu }}_{0}^{C}-{\hat{\mu }}_{1}^{C}\right)<0\\ 0\;,\; & \text{otherwise} \end{array}\right.\;,$$

where

24 $$\;\begin{array}{c} {\hat{\mu }}_{0}^{C}=\frac{\overset{n}{\underset{i=1}{\sum }}C(i)X_{i}I_{Y_{i}=0}}{\overset{n}{\underset{i=1}{\sum }}C(i)I_{Y_{i}=0}}\;\text{and}\;{\hat{\mu }}_{1}^{C}=\frac{\overset{n}{\underset{i=1}{\sum }}C(i)X_{i}I_{Y_{i}=1}}{\overset{n}{\underset{i=1}{\sum }}C(i)I_{Y_{i}=1}} \end{array}$$

are *bootstrap sample means*.

Now, note that with *N*_0_=*n*_0_ fixed, the training data labels *Y*_*i*_, *i*=1,…,*n*, are no longer random. Since all classification rules of interest are invariant to reordering of the training data, we can, without loss of generality, reorder the sample points so that *Y*_*i*_=0 for *i*=1,…,*n*_0_, and *Y*_1_=1 for *i*=*n*_0_+1,…,*n*. Let the same reordering be applied to a given bootstrap vector *C*. The next theorem extends John’s result to the classification error of the bootstrapped LDA classification rule defined by (23).

#### Theorem 1.

Assume that population *Π*_*i*_ is distributed as $N\left(\mu_{i},\sigma_{i}^{2}\right)$, for *i*=0,1. Then the expected error rate of the bootstrap LDA classification rule defined by (23) is given by:

25 $$\begin{array}{c} E\left[\varepsilon_{n}^{C,0}\;|\;N_{0}=n_{0},C\right]\;=\;\Phi (e,f;\rho_{c})+\Phi (-e,-f;\rho_{c})\;, \end{array}$$

where

26 $$\begin{array}{cc} e & =\frac{\mu_{1}-\mu_{0}}{\sqrt{s_{0}\sigma_{0}^{2}+s_{1}\sigma_{1}^{2}}}\;,\;f=\frac{\mu_{0}-\mu_{1}}{\sqrt{(4+s_{0})\sigma_{0}^{2}+s_{1}\sigma_{1}^{2}}}\;,\\ \rho_{c} & =\frac{s_{0}\sigma_{0}^{2}-s_{1}\sigma_{1}^{2}}{\sqrt{\left((4+s_{0})\sigma_{0}^{2}+s_{1}\sigma_{1}^{2}\right)\left(s_{0}\sigma_{0}^{2}+s_{1}\sigma_{1}^{2}\right)}}\;, \end{array}$$

with

27 $$s_{0}\;=\;\frac{\overset{n_{0}}{\underset{i=1}{\sum }}C{\left(i\right)}^{2}}{{\left(\overset{n_{0}}{\underset{i=1}{\sum }}C(i)\right)}^{2}}\;\text{and}\;s_{1}\;=\;\frac{\overset{n_{1}}{\underset{i=1}{\sum }}C{\left(n_{0}+i\right)}^{2}}{{\left(\overset{n_{1}}{\underset{i=1}{\sum }}C(n_{0}+i)\right)}^{2}}\;,$$

The corresponding result for $E\left[\varepsilon_{n}^{C,1}\;|\;N_{0}=n_{0},C\right]$ is obtained by interchanging all indices 0 and 1.

*Proof.* See the Appendix.

It is easy to check that the result in Theorem 1 reduces to the one in (14) and (15) when *C*=**1**_*n*_. Following (16), we can then write

28 $$\begin{array}{cc} E\left[\varepsilon_{n}^{\;C}|C\right] & =\overset{n}{\underset{n_{0}=0}{\sum }}E\left[\varepsilon_{n}^{\;C}|N_{0}=n_{0},C\right]P(N_{0}=n_{0})\\ =\overset{n}{\underset{n_{0}=0}{\sum }}\left(c_{0}E\left[\varepsilon_{n}^{\;C,0}|N_{0}=n_{0},C\right]\right.\\ \;+\left.c_{1}E\left[\varepsilon_{n}^{\;C,1}|N_{0}=n_{0},C\right]\right)P(N_{0}=n_{0}). \end{array}$$

The expected bootstrap error rate $E[{\hat{\varepsilon }}_{n}^{\;\text{boot}}]$ can now be computed via (12).

The weight *w*^∗^ for unbiased bootstrap error estimation can now be computed exactly by means of Equations (11), (12), (14) to (17), (20) to (22), and (25) to (28).

In the special case *σ*_0_=*σ*_1_=*σ* (homoskedasticity), it follows easily from the previous expressions that *E*[*ε*_*n*_], $E[{\hat{\varepsilon }}_{n}^{\;r}]$, and $E[{\hat{\varepsilon }}_{n}^{\;\text{boot}}]$ depend only on the sample size *n* and on the Mahalanobis distance between the populations *δ*=|*μ*_1_−*μ*_0_|/*σ*, and therefore so does the weight *w*^∗^, through (11). Since the optimal (Bayes) classification error in this case is *ε*^∗^=*Φ*(−*δ*/2), there is a one-to-one correspondence between Bayes error and the Mahalanobis distance. Therefore, in the homoskedastic case, the weight *w*^∗^*is a function only of the Bayes error ε*^∗^*and the sample size n*.

**Figure 1:**
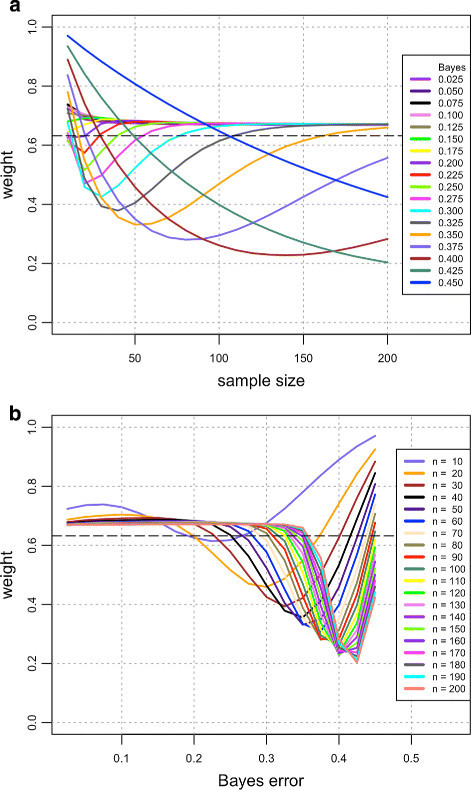
**Univariate case.** Required weight *w*^∗^ for unbiased convex bootstrap estimation plotted against **(a)** sample size and **(b)** Bayes error.

Figure 1 and Table 1 display the value of *w*^∗^ in the homoskedastic case, for several sample sizes and Bayes errors. In order to extend the plots up to *n*=200, it is necessary to approximate $E[{\hat{\varepsilon }}_{n}^{\;\text{boot}}]$ in (12) by a Monte Carlo procedure; this is done by generating *M*=100×*n*^2^ independent random vectors {*C*_*i*_∣*i*=1,…,*M*} and letting $E[{\hat{\varepsilon }}_{n}^{\;\text{boot}}]\approx (1/M)\overset{M}{\underset{i=1}{\sum }}E[\varepsilon_{n}^{\;C_{i}}|C_{i}]$. We find that this value of *M* is large enough to obtain an accurate approximation. All other quantities are computed exactly, as described previously. One can see in Figure 1a that *w*^∗^ varies wildly and can be very far from the heuristic 0.632 weight; however, as the sample size increases, *w*^∗^ appears to settle around an asymptotic fixed value. This asymptotic value is approximately 0.675, being thus slightly larger than 0.632. In addition, Figure 1b allows one to see that convergence to the asymptotic value is faster for smaller Bayes errors. These facts help explain the good performance of the original convex 0.632 bootstrap error estimator with moderate sample sizes and small Bayes errors.

**Table 1:** **Univariate case: required weight** ***w*** ^**∗**^ **for unbiased convex bootstrap estimation**

	***n***=10	***n***=20	***n***=30	***n***=40	***n***=50	***n***=60	***n***=70	***n***=80	***n***=90	***n***=100
*ε*^∗^=0.025	0.724	0.687	0.679	0.675	0.674	0.672	0.671	0.671	0.670	0.670
*ε*^∗^=0.050	0.736	0.696	0.685	0.680	0.678	0.676	0.674	0.673	0.672	0.672
*ε*^∗^=0.075	0.738	0.701	0.689	0.683	0.679	0.677	0.676	0.674	0.674	0.673
*ε*^∗^=0.100	0.729	0.704	0.691	0.684	0.681	0.678	0.677	0.675	0.674	0.673
*ε*^∗^=0.125	0.708	0.701	0.692	0.686	0.682	0.679	0.677	0.676	0.675	0.674
*ε*^∗^=0.150	0.681	0.692	0.693	0.687	0.683	0.680	0.678	0.677	0.676	0.675
*ε*^∗^=0.175	0.646	0.670	0.688	0.687	0.683	0.680	0.678	0.677	0.676	0.675
*ε*^∗^=0.200	0.625	0.631	0.673	0.683	0.683	0.681	0.679	0.677	0.676	0.675
*ε*^∗^=0.225	0.614	0.574	0.639	0.671	0.679	0.680	0.679	0.677	0.676	0.675
*ε*^∗^=0.250	0.617	0.516	0.579	0.635	0.663	0.673	0.676	0.677	0.676	0.675
*ε*^∗^=0.275	0.641	0.470	0.498	0.563	0.617	0.648	0.664	0.671	0.673	0.674
*ε*^∗^=0.300	0.676	0.459	0.425	0.464	0.523	0.577	0.616	0.641	0.656	0.665
*ε*^∗^=0.325	0.724	0.487	0.393	0.379	0.405	0.451	0.502	0.548	0.587	0.614
*ε*^∗^=0.350	0.780	0.549	0.422	0.356	0.331	0.334	0.356	0.389	0.428	0.469
*ε*^∗^=0.375	0.837	0.639	0.505	0.412	0.350	0.310	0.288	0.280	0.282	0.295
*ε*^∗^=0.400	0.890	0.741	0.626	0.533	0.458	0.398	0.350	0.312	0.283	0.261
*ε*^∗^=0.425	0.935	0.842	0.761	0.690	0.627	0.570	0.519	0.474	0.434	0.399
*ε*^∗^=0.450	0.971	0.925	0.884	0.845	0.808	0.772	0.739	0.707	0.676	0.647
	***n*** **=110**	***n*** **=120**	***n*** **=130**	***n*** **=140**	***n*** **=150**	***n*** **=160**	***n*** **=170**	***n*** **=180**	***n*** **=190**	***n*** **=200**
*ε*^∗^=0.025	0.669	0.669	0.669	0.669	0.669	0.669	0.669	0.668	0.668	0.668
*ε*^∗^=0.050	0.671	0.671	0.671	0.671	0.670	0.670	0.670	0.669	0.670	0.669
*ε*^∗^=0.075	0.672	0.672	0.671	0.671	0.671	0.671	0.670	0.670	0.670	0.670
*ε*^∗^=0.100	0.673	0.672	0.672	0.671	0.671	0.671	0.671	0.670	0.670	0.670
*ε*^∗^=0.125	0.673	0.673	0.672	0.672	0.672	0.671	0.671	0.671	0.670	0.670
*ε*^∗^=0.150	0.674	0.673	0.673	0.672	0.672	0.672	0.671	0.671	0.671	0.671
*ε*^∗^=0.175	0.674	0.673	0.673	0.672	0.672	0.672	0.672	0.671	0.671	0.671
*ε*^∗^=0.200	0.674	0.673	0.673	0.673	0.672	0.672	0.672	0.671	0.671	0.671
*ε*^∗^=0.225	0.675	0.674	0.673	0.672	0.672	0.672	0.672	0.672	0.671	0.671
*ε*^∗^=0.250	0.675	0.674	0.673	0.673	0.672	0.672	0.672	0.672	0.671	0.671
*ε*^∗^=0.275	0.674	0.674	0.673	0.673	0.673	0.673	0.672	0.671	0.671	0.671
*ε*^∗^=0.300	0.669	0.671	0.672	0.672	0.672	0.672	0.672	0.672	0.672	0.672
*ε*^∗^=0.325	0.635	0.648	0.657	0.663	0.666	0.668	0.669	0.670	0.671	0.671
*ε*^∗^=0.350	0.508	0.543	0.572	0.597	0.615	0.630	0.642	0.649	0.655	0.660
*ε*^∗^=0.375	0.313	0.337	0.365	0.394	0.425	0.455	0.484	0.511	0.536	0.557
*ε*^∗^=0.400	0.245	0.234	0.229	0.228	0.229	0.235	0.243	0.254	0.268	0.283
*ε*^∗^=0.425	0.367	0.338	0.313	0.290	0.270	0.253	0.238	0.224	0.213	0.203
*ε*^∗^=0.450	0.620	0.594	0.569	0.545	0.522	0.501	0.480	0.461	0.442	0.424

### 4.2 Multivariate case

Assume that population *Π*_*i*_ is distributed as a multivariate Gaussian *N*(*μ*_*i*_,*Σ*), for *i*=0,1. Under these conditions, John obtained in [39] an exact expression for the expectation of the error of the LDA classification rule, defined by (2) to (4), for the case where *N*_0_=*n*_0_ is fixed. This result is stated by Moran in [40] as follows:

29 $$E\left[\varepsilon_{n}^{0}|N_{0}=n_{0}\right]\;=\;P\left(\frac{W_{1}}{W_{2}}>\frac{1-\rho_{e}}{1+\rho_{e}}\right)\;,$$

where *W*_1_ and *W*_2_ are independently distributed as noncentral chi-square variables with *d* degrees of freedom(*d* being the dimensionality) and noncentrality parameters *λ*_1_ and *λ*_2_, with

30 $$\;\begin{array}{cc} \lambda_{1} & \;=\;\frac{n_{0}n_{1}}{2(1+\rho_{e})}\;{\left(\frac{1}{\sqrt{n_{0}+n_{1}}}-\frac{1}{\sqrt{n_{0}+n_{1}+4n_{0}n_{1}}}\right)}^{2}\delta^{2}\;,\\ \lambda_{2} & \;=\;\frac{n_{0}n_{1}}{2(1-\rho_{e})}\;{\left(\frac{1}{\sqrt{n_{0}+n_{1}}}+\frac{1}{\sqrt{n_{0}+n_{1}+4n_{0}n_{1}}}\right)}^{2}\delta^{2}\;,\\ \rho_{e} & \;=\;\frac{n_{1}-n_{0}}{\sqrt{\left(n_{0}+n_{1})(n_{0}+n_{1}+4n_{0}n_{1}\right)}}\;, \end{array}$$

where *δ*^2^ = (*μ*_1_−*μ*_0_)^*T*^*Σ*^−1^(*μ*_1_−*μ*_0_) is the squared Mahalanobis distance between the populations. The corresponding result for $E[\varepsilon_{n}^{1}|N_{0}=n_{0}]$ is obtained by interchanging *n*_0_ and *n*_1_. The expected true error rate can then be found by using (16).

Moran also provided the following expression for the expectation of the resubstitution error estimator in the multivariate case, for fixed *N*_0_=*n*_0_[40]:

31 $$E\left[{\hat{\varepsilon }}_{n}^{\;r,0}|N_{0}=n_{0}\right]\;=\;P\left(\frac{W_{3}}{W_{4}}>\frac{1-\rho_{r}}{1+\rho_{r}}\right)\;,$$

where *W*_3_ and *W*_4_ are independently distributed as noncentral chi-square variables with *d* degrees of freedom and noncentrality parameters *λ*_3_ and *λ*_4_, with

32 $$\;\begin{array}{cc} \lambda_{3} & =\frac{n_{0}n_{1}}{2(1+\rho_{r})}\;{\left(\frac{1}{\sqrt{n_{0}+n_{1}}}-\frac{1}{\sqrt{n_{0}-3n_{1}+4n_{0}n_{1}}}\right)}^{2}\delta^{2}\;,\\ \lambda_{4} & =\frac{n_{0}n_{1}}{2(1-\rho_{r})}\;{\left(\frac{1}{\sqrt{n_{0}+n_{1}}}+\frac{1}{\sqrt{n_{0}-3n_{1}+4n_{0}n_{1}}}\right)}^{2}\delta^{2}\;,\\ \rho_{r} & =-\sqrt{\frac{n_{0}+n_{1}}{n_{0}-3n_{1}+4n_{0}n_{1}}}\;, \end{array}$$

The corresponding result for $E[{\hat{\varepsilon }}_{n}^{\;r,1}]$ is obtained by interchanging *n*_0_ and *n*_1_. The expected resubstitution error rate can then be found by using (22).

The bootstrap LDA classifier in the multivariate case is given by

33 $$\psi_{n}^{C}(X)=\left\{\begin{array}{cc} 1\;, & \;\text{if}\;{\left(X-\frac{{\hat{\mu }}_{0}^{C}+{\hat{\mu }}_{1}^{C}}{2}\right)}^{T}\Sigma^{-1}\left({\hat{\mu }}_{0}^{C}-{\hat{\mu }}_{1}^{C}\right)<0\\ 0\;, & \;\text{otherwise} \end{array}\right.\;,$$

where ${\hat{\mu }}_{0}^{C}$ and ${\hat{\mu }}_{1}^{C}$ are defined in (24). The next theorem generalizes John’s result for the multivariate classification error to the case of the bootstrapped LDA classification rule.

#### Theorem 2.

Assume that population *Π*_*i*_ is distributed as *N*(*μ*_*i*_,*Σ*), for *i*=0,1. Then, the expected error rate of the bootstrap LDA classification rule defined by (33) is given by

34 $$E\left[\varepsilon_{n}^{C,0}\;|\;N_{0}=n_{0},C\right]\;=\;P\left(\frac{W_{5}}{W_{6}}>\frac{1-\rho_{c}}{1+\rho_{c}}\right)\;,$$

where *W*_5_ and *W*_6_ are independently distributed as noncentral chi-square variables with *d* degrees of freedom and noncentrality parameters *λ*_5_ and *λ*_6_, with

35 $$\begin{array}{cc} \lambda_{5} & \;=\;\frac{1}{2(1+\rho_{c})}\;{\left(\frac{1}{\sqrt{s_{0}+s_{1}}}-\frac{1}{\sqrt{s_{0}+s_{1}+4}}\right)}^{2}\delta^{2}\;,\\ \lambda_{6} & \;=\;\frac{1}{2(1-\rho_{c})}\;{\left(\frac{1}{\sqrt{s_{0}+s_{1}}}+\frac{1}{\sqrt{s_{0}+s_{1}+4}}\right)}^{2}\delta^{2}\;,\\ \rho_{c} & \;=\;\frac{s_{0}-s_{1}}{\sqrt{\left(s_{0}+s_{1})(s_{0}+s_{1}+4\right)}}\;, \end{array}$$

where *s*_0_ and *s*_1_ are defined in (27). The corresponding result for $E[\varepsilon_{n}^{C,1}\;|\;N_{0}=n_{0},C]$ is obtained by interchanging *s*_0_ and *s*_1_.

*Proof.* See the Appendix.

It is easy to check that the result in Theorem 2 reduces to the one in (29) and (30) when *C*=**1**_*n*_.

As in the univariate case, Theorem 2 can be used in conjunction with Equations (12) and (28) to compute $E[{\hat{\varepsilon }}_{n}^{\;\text{boot}}]$.

The weight *w*^∗^ for unbiased bootstrap error estimation can now be computed exactly by means of Equations (11), (12), (16) to (17), (22), (28), (29) to (32), and (34) to (35).

An issue that arises in the multivariate case is the computation of the probabilities in (29), (31), and (34). This computation is very difficult since it involves the ratio of noncentral chi-square random variables, which has a doubly noncentral F distribution. Computation of this distribution is a hard problem. Moran proposes in [40] a complex procedure, based on work by Price [53], to compute this probability, which only applies to even dimensionality *d*. We employ a simpler procedure, namely, the Imhof-Pearson three-moment method, which is applicable to even and odd dimensionality [41]. This consists of approximating a noncentral $\chi_{d}^{2}(\lambda )$ random variable with a central $\chi_{h}^{2}$ random variable, by equating the first three moments of their distributions. This approach was also employed in [52], where it was found to be very accurate. To fix ideas, we consider (29). The Imhof-Pearson three-moment approximation is given by

36 $$E\left[\varepsilon_{n}^{0}\right]\;=\;P\left(\frac{W_{1}}{W_{2}}>\frac{1-\rho_{e}}{1+\rho_{e}}\right)\;≃\;P\left(\chi_{h}^{2}>y\right)\;,$$

where $\chi_{h}^{2}$ is a central chi-square random variable with *h* degrees of freedom, with

37 $$\begin{array}{cc} h & \;=\;\frac{c_{2}^{3}}{c_{3}^{2}}\;,\\ y & \;=\;h-c_{1}\;\sqrt{\frac{h}{c_{2}}}\;, \end{array}$$

and

38 $$\begin{array}{cc} c_{i} & ={\left(1+\rho_{e}\right)}^{i}(d+i\lambda_{1})\;+\;{\left(-1\right)}^{i}{\left(1-\rho_{e}\right)}^{i}(d+i\lambda_{2}),\\ i & =1,2,3. \end{array}$$

The approximation is valid only for *c*_3_>0 [41]. If *c*_3_<0, one uses the approximation

39 $$E\left[\varepsilon_{n}^{0}\right]\;=\;P\left(\frac{W_{1}}{W_{2}}>\frac{1-\rho_{e}}{1+\rho_{e}}\right)\;≃\;P\left(\chi_{h}^{2}<y\right)\;,$$

where *h* and *y* are as in (37), and

40 $$\begin{array}{cc} c_{i} & ={\left(-1\right)}^{i}{\left(1+\rho_{e}\right)}^{i}(d+i\lambda_{1})\;+\;{\left(1-\rho_{e}\right)}^{i}(d+i\lambda_{2}),\\ i & =1,2,3\;. \end{array}$$

The same approximation method applies to (31) and (34) by substituting the appropriate values.

As in the univariate case, the assumption of a common covariance matrix *Σ* makes the expectations *E*[*ε*_*n*_], $E[{\hat{\varepsilon }}_{n}^{\;r}]$, and $E[{\hat{\varepsilon }}_{n}^{\;\text{boot}}]$ and thus also the weight *w*^∗^, functions only of *n* and *δ*. Since *ε*^∗^=*Φ*(−*δ*/2), this means that the weight *w*^∗^ is a function only of the Bayes error *ε*^∗^ and the sample size *n*.

**Figure 2:**
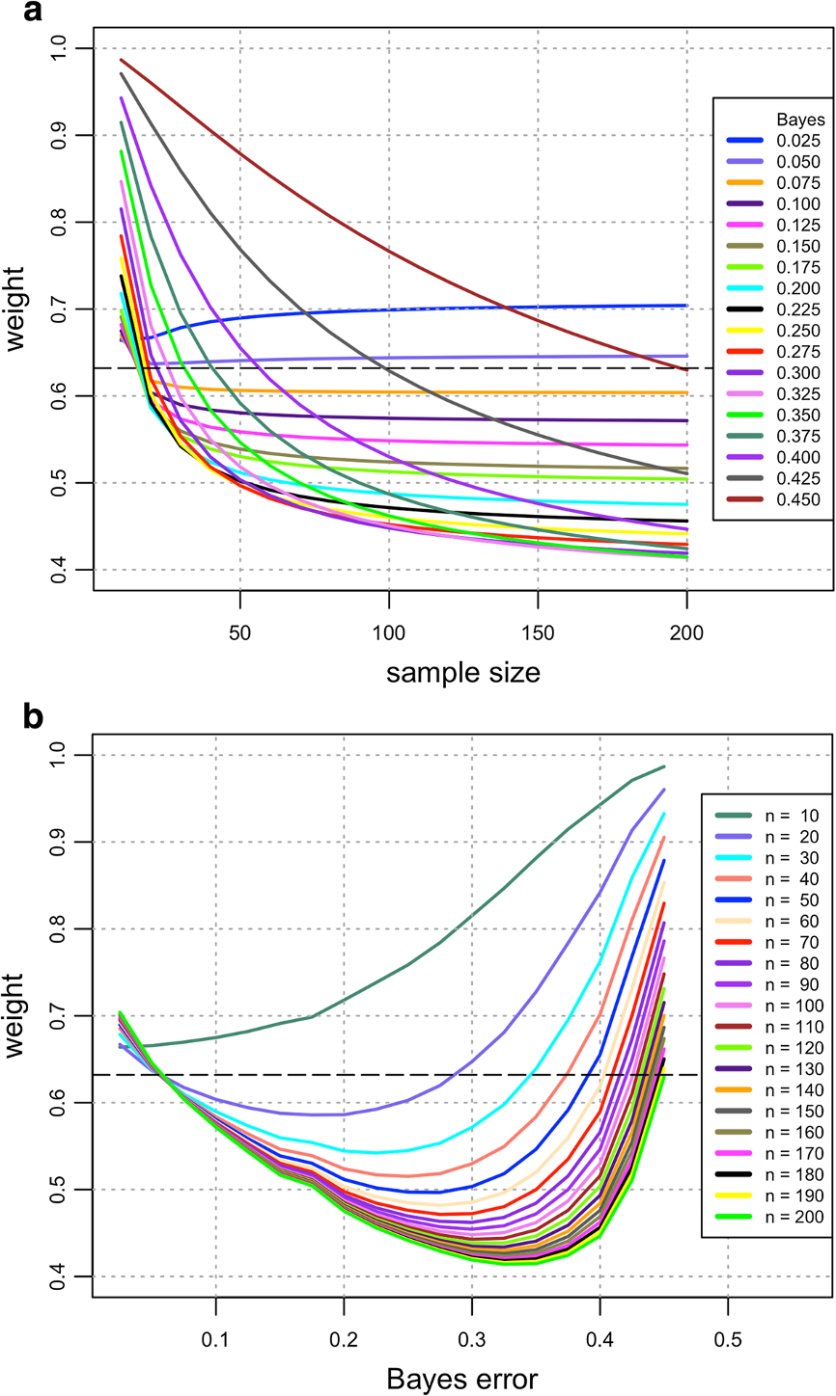
**Bivariate case.** Required weight *w*^∗^ for unbiased convex bootstrap estimation plotted against **(a)** sample size and **(b)** Bayes error.

Figure 2 and Table 2 display the value of *w*^∗^ computed with the previous expressions in this section, for several sample sizes and Bayes errors. As in the univariate case, $E[{\hat{\varepsilon }}_{n}^{\;\text{boot}}]$ in (12) is approximated by a Monte Carlo procedure, with the same number *M*=100×*n*^2^ of MC vectors. All other quantities are computed exactly, as described previously, save for the Imhof-Pearson approximation. We can see in Figure 2 that there is considerable variation in the value of *w*^∗^ and it can be far from the heuristic 0.632 weight; however, as the sample size increases, *w*^∗^ appears to settle around an asymptotic fixed value. In contrast to the univariate case, these asymptotic values here appear to be strongly dependent on the Bayes error and are significantly smaller than the heuristic 0.632 except for very small Bayes errors. As in the univariate case, convergence to the apparent asymptotic value is faster for smaller Bayes errors. These facts again help explain the good performance of the original convex 0.632 bootstrap error estimator for moderate sample sizes and small Bayes errors.

**Table 2:** **Bivariate case: required weight** ***w*** ^**∗**^ **for unbiased convex bootstrap estimation**

	***n***=10	***n***=20	***n***=30	***n***=40	***n***=50	***n***=60	***n***=70	***n***=80	***n***=90	***n***=100
*ε*^∗^=0.025	0.664	0.667	0.679	0.685	0.690	0.693	0.695	0.697	0.698	0.699
*ε*^∗^=0.050	0.666	0.637	0.638	0.639	0.641	0.642	0.642	0.643	0.644	0.644
*ε*^∗^=0.075	0.670	0.617	0.610	0.608	0.606	0.606	0.605	0.605	0.605	0.605
*ε*^∗^=0.100	0.675	0.604	0.590	0.584	0.581	0.578	0.577	0.576	0.575	0.574
*ε*^∗^=0.125	0.682	0.594	0.573	0.564	0.559	0.555	0.553	0.551	0.550	0.548
*ε*^∗^=0.150	0.691	0.588	0.560	0.547	0.539	0.534	0.530	0.528	0.526	0.524
*ε*^∗^=0.175	0.699	0.586	0.554	0.539	0.530	0.524	0.520	0.517	0.515	0.513
*ε*^∗^=0.200	0.718	0.586	0.544	0.524	0.512	0.504	0.498	0.493	0.490	0.487
*ε*^∗^=0.225	0.738	0.592	0.542	0.517	0.502	0.492	0.485	0.479	0.475	0.471
*ε*^∗^=0.250	0.759	0.603	0.545	0.515	0.497	0.485	0.476	0.469	0.464	0.460
*ε*^∗^=0.275	0.784	0.620	0.553	0.518	0.497	0.482	0.471	0.463	0.457	0.452
*ε*^∗^=0.300	0.815	0.647	0.572	0.530	0.503	0.485	0.472	0.462	0.454	0.448
*ε*^∗^=0.325	0.847	0.681	0.598	0.550	0.518	0.496	0.480	0.468	0.458	0.450
*ε*^∗^=0.350	0.882	0.728	0.639	0.584	0.546	0.520	0.500	0.484	0.472	0.462
*ε*^∗^=0.375	0.915	0.784	0.695	0.635	0.592	0.560	0.535	0.516	0.500	0.487
*ε*^∗^=0.400	0.943	0.842	0.763	0.702	0.655	0.619	0.590	0.566	0.546	0.530
*ε*^∗^=0.425	0.971	0.914	0.859	0.811	0.769	0.732	0.701	0.673	0.650	0.629
*ε*^∗^=0.450	0.987	0.960	0.933	0.905	0.879	0.853	0.830	0.807	0.786	0.766
	***n*** **=110**	***n*** **=120**	***n*** **=130**	***n*** **=140**	***n*** **=150**	***n*** **=160**	***n*** **=170**	***n*** **=180**	***n*** **=190**	***n*** **=200**
*ε*^∗^=0.025	0.700	0.701	0.701	0.702	0.702	0.703	0.703	0.704	0.704	0.704
*ε*^∗^=0.050	0.644	0.645	0.645	0.645	0.645	0.645	0.645	0.646	0.646	0.646
*ε*^∗^=0.075	0.604	0.604	0.604	0.604	0.604	0.604	0.604	0.604	0.604	0.604
*ε*^∗^=0.100	0.574	0.573	0.573	0.573	0.573	0.572	0.572	0.572	0.572	0.572
*ε*^∗^=0.125	0.548	0.547	0.546	0.546	0.545	0.545	0.544	0.544	0.544	0.543
*ε*^∗^=0.150	0.523	0.522	0.521	0.520	0.519	0.518	0.518	0.517	0.517	0.517
*ε*^∗^=0.175	0.511	0.510	0.509	0.508	0.507	0.506	0.506	0.505	0.505	0.504
*ε*^∗^=0.200	0.485	0.483	0.482	0.480	0.479	0.478	0.477	0.477	0.476	0.475
*ε*^∗^=0.225	0.469	0.466	0.464	0.463	0.461	0.460	0.459	0.458	0.457	0.456
*ε*^∗^=0.250	0.457	0.454	0.452	0.449	0.448	0.446	0.445	0.443	0.442	0.441
*ε*^∗^=0.275	0.448	0.444	0.442	0.439	0.437	0.435	0.433	0.432	0.430	0.429
*ε*^∗^=0.300	0.443	0.438	0.435	0.432	0.429	0.426	0.424	0.422	0.420	0.419
*ε*^∗^=0.325	0.444	0.439	0.434	0.430	0.426	0.423	0.421	0.418	0.416	0.414
*ε*^∗^=0.350	0.454	0.447	0.441	0.435	0.431	0.427	0.423	0.420	0.417	0.415
*ε*^∗^=0.375	0.476	0.467	0.459	0.452	0.446	0.441	0.436	0.432	0.428	0.424
*ε*^∗^=0.400	0.516	0.504	0.493	0.484	0.476	0.469	0.462	0.457	0.451	0.447
*ε*^∗^=0.425	0.611	0.594	0.580	0.567	0.555	0.544	0.535	0.526	0.518	0.511
*ε*^∗^=0.450	0.748	0.731	0.715	0.700	0.687	0.674	0.662	0.650	0.640	0.630

## 5Gene expression classification example

Here we demonstrate the application of the previous theory in comparing the performance of the bootstrap error estimator using the optimal weight versus the use of the fixed *w*=0.632 weight, using gene expression data from the well-known breast cancer classification study in [42], which analyzed expression profiles from 295 tumor specimens, divided into *N*_0_=115 specimens belonging to the ‘good-prognosis’ population (class 1 here) and *N*_1_=180 specimens belonging to the ‘poor-prognosis’ population (class 0).

**Figure 3:**
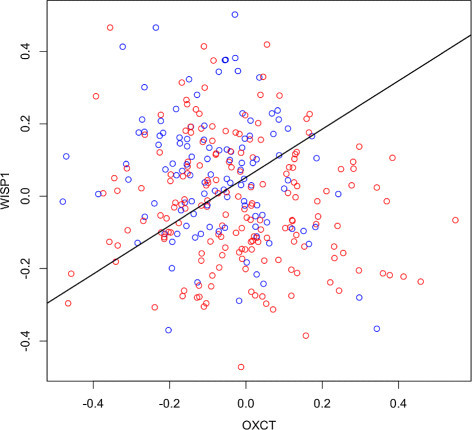
**Data used in the gene expression experiment.** The plot shows the optimal (linear) classifier superimposed on the sample for the genes OXCT and WISP1, from the breast cancer study in [42]. We can see that both populations are approximately Gaussian with equal dispersion. Bad prognosis = red. Good prognosis = blue.

Our experiment was set up in the following way. We selected two genes among the previously published 70-gene prognosis profile [43]. These genes were selected for their approximate homoskedastic Gaussian distributions (see Figure 3). Since the real prior probabilities *c*_0_ and *c*_1_ for the good- and poor-prognosis populations are unknown, we assumed three different scenarios corresponding to *c*_0_=1/3, *c*_0_=1/2, and *c*_0_=2/3 and *downsampled* randomly one or the other set of specimens to obtain new sample sizes (90,180), (115,115), and (115,68), respectively, so as to reflect the assumed prior probabilities. In each of the three cases, we then drew 2,000 random samples of size *n*=30 from the pooled data, computed for each the true error, resubstitution, basic bootstrap, and convex bootstrap error rates. Bias and root-mean-square (RMS) error for each estimator were estimated by averaging over the 2,000 repetitions. We considered both the fixed 0.632 weight and the optimal weight prescribed by our analysis. For the latter, we estimated for each value of *c*_0_ the Bayes error using the full data set and read off Table 2 the optimal weight corresponding to the estimated Bayes error and sample size *n*=30. The results are displayed in Table 3. Despite the approximate nature of the results, given that the simulated training samples are not independent from each other, we can see that the bias and RMS were always smaller for the estimator using the optimal weight than using the fixed 0.632 weight (all bootstrap estimators vastly outperforming resubstitution).

**Table 3:** **Bias and RMS of estimators considered in the experiment with expression data from genes ‘OXCT’ and ‘WISP1’**

***c***_0_	n	***ε*** ^∗^	***E***[***ε***_***n***_]	Resub		Basic boot		Opt boot		0.632 boot	
				Bias	RMS	Bias	RMS	Bias	RMS	Bias	RMS
0.33	30	0.4043	0.4206	−0.0702	0.1061	0.0008	0.0820	−0.0161	0.0803	−0.0253	0.0817
0.50	30	0.3969	0.4266	−0.0719	0.1060	0.0072	0.0830	−0.0116	0.0798	−0.0219	0.0806
0.67	30	0.3893	0.4131	−0.0914	0.1185	−0.0181	0.0878	−0.0355	0.0885	−0.0451	0.0909

Also displayed are the assumed values for the prior probability *c*_0_, sample size *n*, the estimated value of the Bayes error *ε*^∗^, and the expected classification error *E*[ *ε*_n_].

## 6Conclusions

Exact expressions were derived for the required weight for unbiased convex bootstrap error estimation in the finite sample case, for linear discriminant analysis of Gaussian populations. The results not only provide the practitioner with a recommendation of what weight to use given the sample size and problem difficulty, but also offer insight into the choice of the 0.632 weight for the classic 0.632 bootstrap error estimator. It was observed that the required weight for unbiasedness can deviate significantly from the 0.632 weight, particularly in the multivariate case, where the required weight for unbiasedness appears to settle on an asymptotic value that is strongly dependent on the Bayes error, being as a rule smaller than 0.632. The results were illustrated by application to gene expression data from a well-known breast cancer study.

## 7Appendix

**Proof of Theorem 1**

Following the same technique used in [40], we write

41 $$\begin{array}{ll} \;E\left[\varepsilon_{C}^{0}\;|\;C\right] & =P\left(\psi_{n}^{C}(X)=1|X\in \Pi_{0},C\right)\;\\ \;=\;P\left({\hat{\mu }}_{1}^{C}>{\hat{\mu }}_{0}^{C},X>\frac{{\hat{\mu }}_{0}^{C}+{\hat{\mu }}_{1}^{C}}{2}|X\in \Pi_{0},C\right)\;\\ \;+P\left({\hat{\mu }}_{1}^{C}\leq {\hat{\mu }}_{0}^{C},X\leq \frac{{\hat{\mu }}_{0}^{C}+{\hat{\mu }}_{1}^{C}}{2}|X\in \Pi_{0},C\right)\;\\ \;=\;P(\text{UV}>0|X\in \Pi_{0},C)\;,\; \end{array}$$

where $U={\hat{\mu }}_{1}^{C}-{\hat{\mu }}_{0}^{C}$ and $V=X-\frac{{\hat{\mu }}_{0}^{C}+{\hat{\mu }}_{1}^{C}}{2}$. From (303), it is clear that, given *C*, ${\hat{\mu }}_{0}^{C}$ and ${\hat{\mu }}_{1}^{C}$ are independent Gaussian random variables, such that ${\hat{\mu }}_{i}^{C}∼N(\mu_{i},s_{i}\sigma_{i}^{2})$, for *i*=0,1, where *s*_1_ and *s*_2_ are defined in (27). It follows that *U* and *V* are jointly Gaussian random variables, with the following parameters:

42 $$\begin{array}{l} \;E[\;U\;|\;X\;\in \;\Pi_{0},C]\;=\;\mu_{1}\;-\;\mu_{0},\;\text{Var}(U\;|\;X\;\in \;\Pi_{0},C)\;=\;s_{0}\sigma_{0}^{2}\;+\;s_{1}\sigma_{1}^{2},\;\\ \;E[V|X\in \Pi_{0},C]=\frac{\mu_{0}-\mu_{1}}{2},\;\\ \;\text{Var}(V|X\in \Pi_{0},C)=\left(1+\frac{s_{0}}{4}\right)\sigma_{0}^{2}+\frac{s_{1}}{4}\sigma_{1}^{2}\;,\;\\ \;\text{Cov}(U,V|X\in \Pi_{0},C)=\frac{s_{0}\sigma_{0}^{2}-s_{1}\sigma_{1}^{2}}{2}\;.\; \end{array}$$

The result then follows after some algebraic manipulation. By symmetry, to obtain $E[\varepsilon_{C}^{1}\;|\;C]$, one needs only to interchange all indices 0 and 1. □

**Proof of Theorem 2**

Following the same technique used in [32], we write

43 $$\;\begin{array}{cc} E[\varepsilon_{C}^{0}\;|\;C] & =P(\psi_{n}^{C}(X)=1|X\in \Pi_{0},C)\\ =P\left({\left({\hat{\mu }}_{1}^{C}-{\hat{\mu }}_{0}^{C}\right)}^{T}\Sigma^{-1}\left(X-\frac{{\hat{\mu }}_{0}^{C}+{\hat{\mu }}_{1}^{C}}{2}\right)\right.\\ >\;\;\left.0|X\in \Pi_{0},C\right)=P\left(U^{T}V>0|X\in \Pi_{0},C\right)\\ =P({\left(U+V\right)}^{T}(U+V)-{\left(U-V\right)}^{T}(U-V)\\ >0|X\in \Pi_{0},C)\\ =P\left(\frac{{\left(U+V\right)}^{T}(U+V)}{{\left(U-V\right)}^{T}(U-V)}>1|X\in \Pi_{0},C\right)\;, \end{array}$$

where $U={\left(s_{0}+s_{1}\right)}^{-\frac{1}{2}}\Sigma^{-\frac{1}{2}}({\hat{\mu }}_{1}^{C}-{\hat{\mu }}_{0}^{C})$ and $V=2\;{\left(s_{0}+s_{1}+4\right)}^{-\frac{1}{2}}\Sigma^{-\frac{1}{2}}\left(X-\frac{{\hat{\mu }}_{0}^{C}+{\hat{\mu }}_{1}^{C}}{2}\right)$. It can be readily checked that *U*+*V* and *U*−*V* are independent Gaussian random vectors, such that

44 $$\;\begin{array}{cc} E\left[U+V|X\in \Pi_{0},C\right] & =\left[{\left(s_{0}+s_{1}\right)}^{-\frac{1}{2}}-{\left(s_{0}+s_{1}+4\right)}^{-\frac{1}{2}}\right]\\ \;\times \Sigma^{-1/2}(\mu_{1}-\mu_{0}),\\ E\left[U-V|X\in \Pi_{0},C\right] & =\left[{\left(s_{0}+s_{1}\right)}^{-\frac{1}{2}}+{\left(s_{0}+s_{1}+4\right)}^{-\frac{1}{2}}\right]\\ \;\times \Sigma^{-1/2}(\mu_{1}-\mu_{0})\;,\\ \Sigma_{U+V}|X\in \Pi_{0},C & =2(1+\rho_{c})I\;,\;\Sigma_{U-V}|X\in \Pi_{0},\\ C & =2(1-\rho_{c})I\;, \end{array}$$

where *ρ*_*c*_ is defined as in (35) and *I* denotes the identity matrix of dimension *d*. It follows that

45 $$\begin{array}{cc} W_{5} & =\frac{1}{2(1+\rho_{c})}{\left(U+V\right)}^{T}(U+V)\;,\\ W_{6} & =\frac{1}{2(1-\rho_{c})}{\left(U-V\right)}^{T}(U-V) \end{array}$$

are independent noncentralchi-squared random variables with *d* degrees of freedom and noncentrality parameters *λ*_5_ and *λ*_6_ defined in (35). The result then follows from (62). Following along the same lines, one can show that $E[\varepsilon_{C}^{1}\;|\;C]$ is obtained by interchanging *s*_0_ and *s*_1_ in the result for $E[\varepsilon_{C}^{0}\;|\;C]$ (the details are omitted for brevity). □

## Authors’ original submitted files for images

Below are the links to the authors’ original submitted files for images. Authors’ original file for figure 1 Authors’ original file for figure 2 Authors’ original file for figure 3 Authors’ original file for figure 4 Authors’ original file for figure 5
