# Supplementary figures and images for: Unbiased bootstrap error estimation for linear discriminant analysis
Source: EURASIP J Bioinform Syst Biol. 2014 Oct 3;2014:15. doi: 10.1186/s13637-014-0015-0 (PMC5270504; doi:10.1186/s13637-014-0015-0)

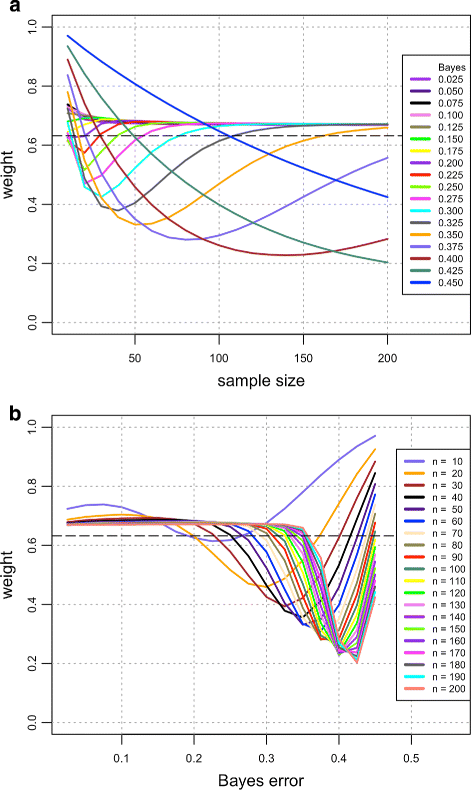

Supplement: Supplementary file 1 — Authors’ original file for figure 1 [file 13637_2014_15_MOESM1_ESM.gif]

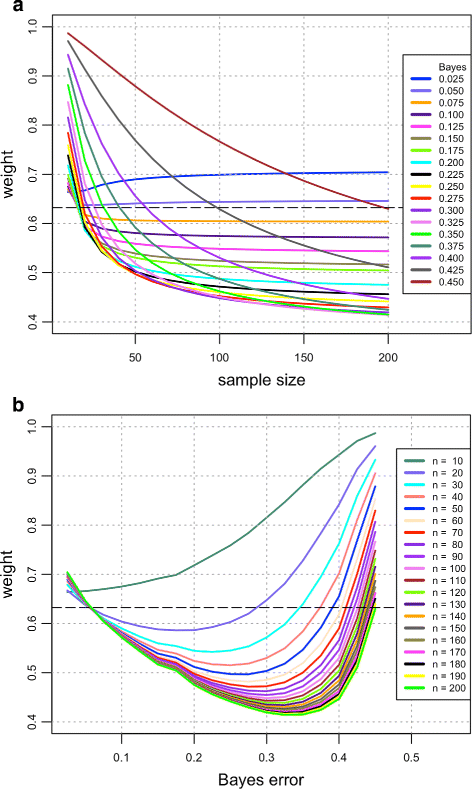

Supplement: Supplementary file 2 — Authors’ original file for figure 2 [file 13637_2014_15_MOESM2_ESM.gif]

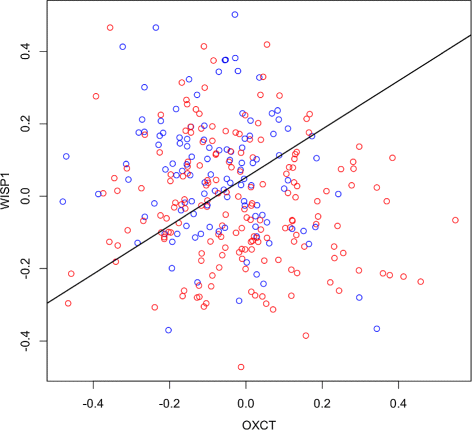

Supplement: Supplementary file 3 — Authors’ original file for figure 3 [file 13637_2014_15_MOESM3_ESM.gif]

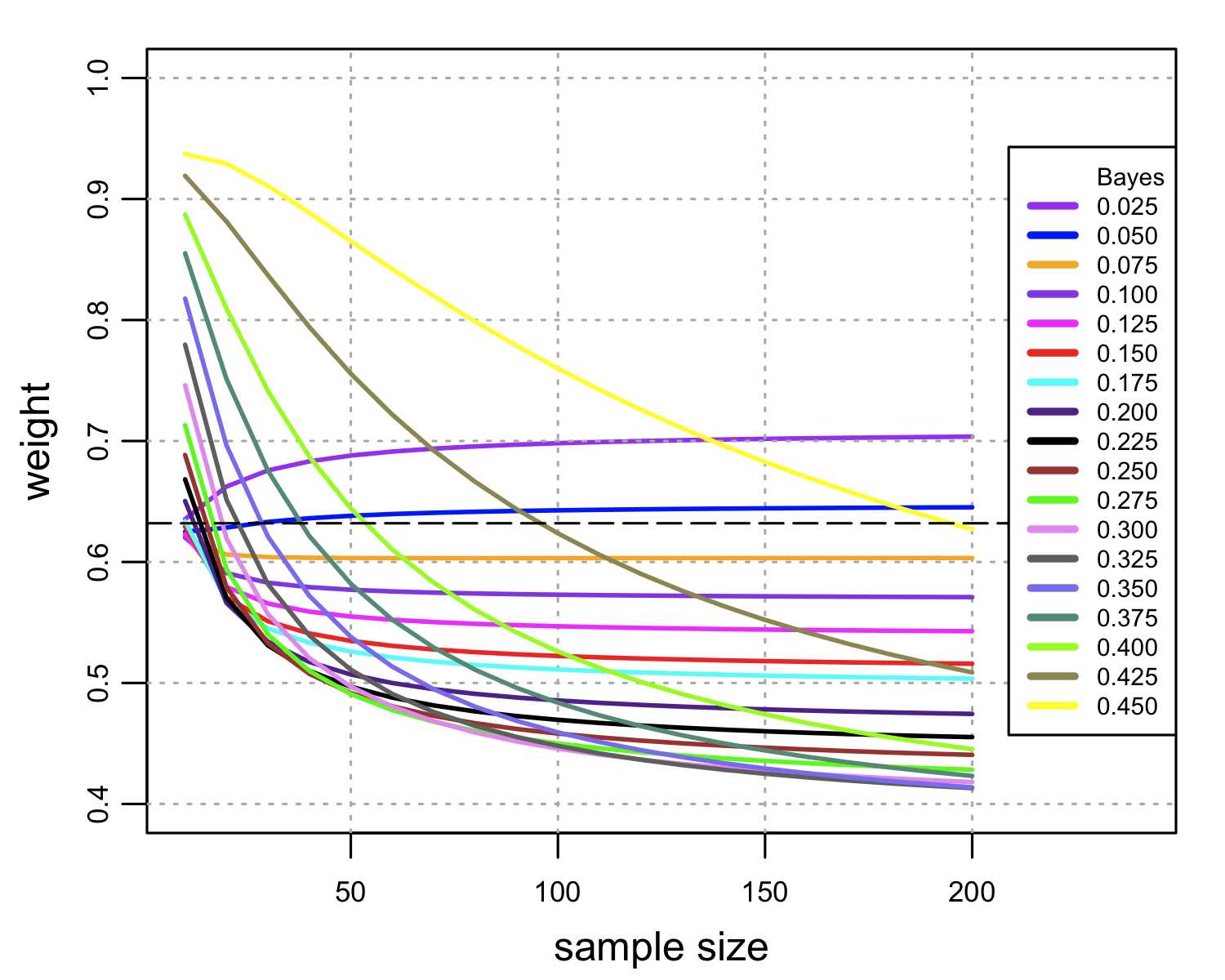

Supplement: Supplementary file 4 — Authors’ original file for figure 4 [file 13637_2014_15_MOESM4_ESM.png]

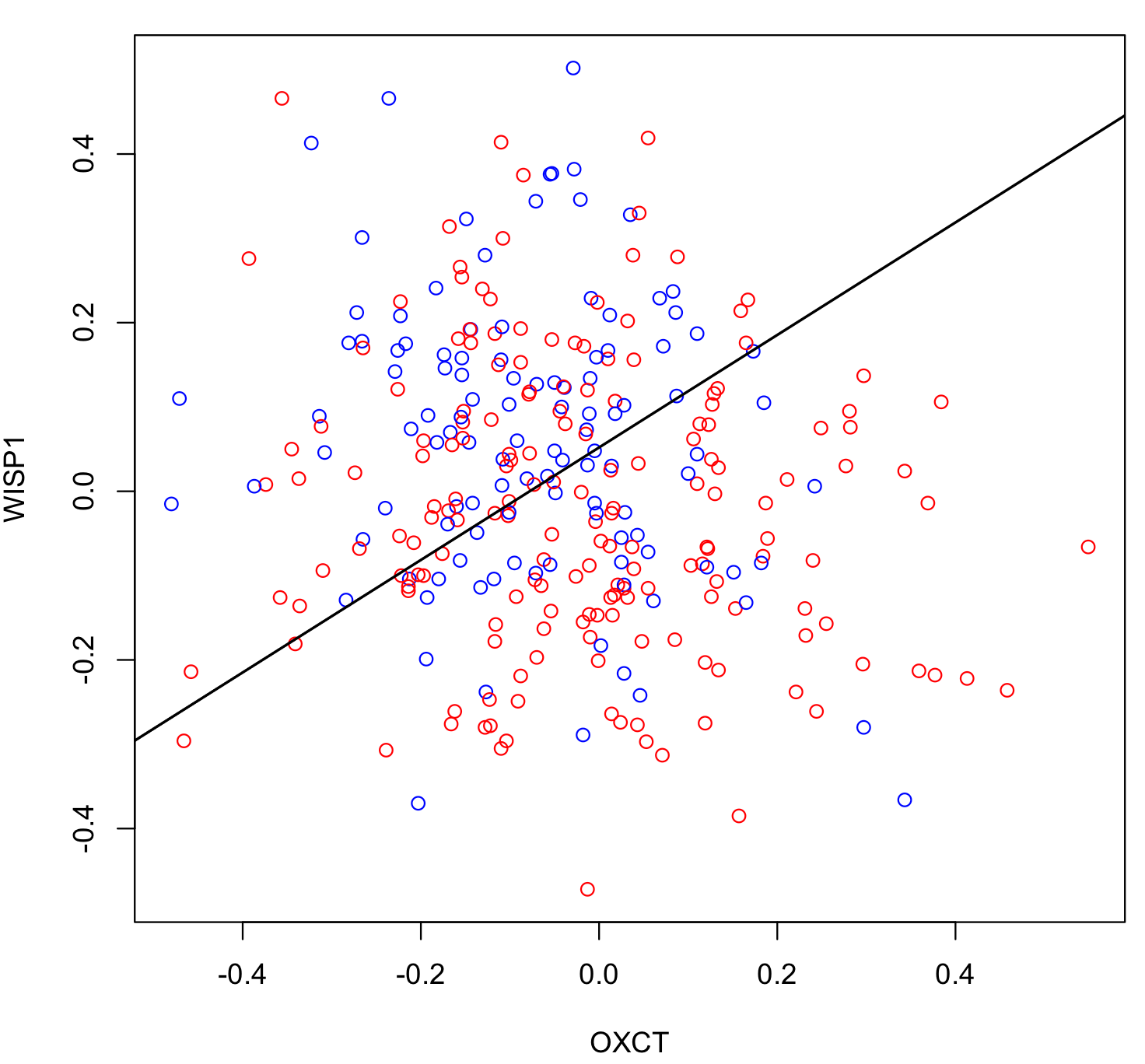

Supplement: Supplementary file 5 — Authors’ original file for figure 5 [file 13637_2014_15_MOESM5_ESM.png]
